# A General Strategy for the Preparation of Carbon Nanotubes and Graphene Oxide Decorated with PdO Nanoparticles in Water

**DOI:** 10.3390/molecules15074679

**Published:** 2010-07-02

**Authors:** Hongkun He, Chao Gao

**Affiliations:** MOE Key Laboratory of Macromolecular Synthesis and Functionalization, Department of Polymer Science and Engineering, Zhejiang University, 38 Zheda Road, Hangzhou 310027, China

**Keywords:** palladium oxide, nanoparticles, carbon nanotubes, graphene, nanohybrids, catalysis

## Abstract

The preparation of carbon nanotube (CNT)/PdO nanoparticles and graphene oxide (GO)/PdO nanoparticle hybrids via a general aqueous solution strategy is reported. The PdO nanoparticles are generated *in situ* on the CNTs and GO by a one-step “green” synthetic approach in aqueous Pd(NO_3_)_2_ solution under ambient conditions without adding any additional chemicals. The production of PdO is confirmed by energy dispersive X-ray spectroscopy, X-ray diffraction, X-ray photoelectron spectroscopy, Raman spectroscopy, and thermal gravimetric analysis. The morphologies of the resulting CNT/PdO and GO/PdO nanohybrids are characterized by transmission and/or scanning transmission electron microscopy. PdO nanoparticles with an average size of 2–3 nm in diameter are decorated evenly along the surfaces of CNTs and GO. This synthesis strategy is demonstrated to be compatible for 1) CNTs with different modifications, including pristine, oxidized, and polymer-functionalized CNTs; 2) different types of CNTs, including single-walled carbon nanotubes (SWCNTs), double-walled carbon nanotubes (DWCNTs), and multiwalled carbon nanotubes (MWCNTs); and 3) different shapes of carbon materials, including tubular CNTs and planar GO. The as-prepared CNT/PdO and GO/PdO nanohybrids can be transformed into CNT/Pd and GO/Pd nanohybrids by reduction with NaBH_4_, and can then be used as a heterogeneous catalyst in the catalytic reduction of 4-nitrophenol.

## 1. Introduction

Nanoparticles (NPs) exhibit greater catalytic efficiency per gram than bulk catalysts due to their higher surface-to-volume ratio [1]. The use of metal nanoparticles (MNPs) in catalysis has fueled intense interest in the development of nanocatalyst over the past decade [2,3]. In comparison with the extensive study of transition metal nanoparticles, the research on their oxide counterparts is quite limited. Particularly, palladium (Pd) and palladium oxide (PdO) are attractive transition metal and metal oxide owing to their unusual physical and chemical properties, which find applications in the catalysis of methane combustion [4], CO oxidation [5,6], methanol oxidation [7], and so forth. A myriad of papers have appeared involving the synthesis of Pd NPs, while there are only a few scattered reports concerning the synthesis of PdO NPs [8,9].

Catalyst support is a key issue in the promotion of heterogeneous catalysis, since the supporter has significant influence on the morphology, electronic state and catalytic activity of supported NPs [10]. To date, various materials have been used to fabricate supported PdO catalysts. Examples include Al_2_O_3_ [11], SnO_2_ [12], zeolites [13], *etc*. However, almost all of these previous synthesis processes employed the impregnation technique, which includes incipient wetness impregnation of supports with palladium precursor solutions followed by the calcination at an elevated temperature to completely decompose the palladium precursor. This impregnation technique is complex and tedious to perform, and is not suitable for some supports that cannot tolerate high temperatures. It is also worth mentioning that unlike most other transition-metal oxides that are expected to interact only weakly with alkanes, PdO is found to be more active than metallic Pd in the catalytic combustion of alkanes [14,15]. Therefore, it is necessary and worthwhile to develop a convenient method for the synthesis of supported PdO catalyst.

Besides the aforesaid catalyst supports, carbon nanotubes (CNTs) and graphene are promising candidates due to their extraordinary structural, electronic, adsorption, mechanical and thermal properties [16,17]. CNTs are tubular carbon materials while graphene sheets consist of planar honeycomb lattice of carbon atoms. Graphene oxide (GO) is oxidized graphene sheet and recently received much attention. Despite there being a plethora of literature involving the deposition of MNPs or metal oxide NPs on CNTs [18,19,20] and GO [21] via electrochemical, chemical or physical methods, no report has yet appeared about depositing PdO on CNTs and GO.

Herein we reported the preparation of PdO-decorated CNTs and GO for the first time. We propose an aqueous solution strategy for the synthesis of PdO NPs on carbon nanomaterials according to the “green chemistry” principles, which possesses several striking merits. Firstly, the synthesis is conducted at room temperature, which differs essentially from the traditional calcination methods [11,12,13] since no high temperature is required. Secondly, the reaction proceeds in water instead of polluting or toxic organic solvents, and no additional reagents were added, making it an environmentally friendly and economically favorable process. Thirdly, the one-step synthesis is easily conducted in mild conditions, facilitating the simple and scalable production of PdO-contained nanohybrids. This aqueous solution strategy is also a general strategy that is useful for various kinds of carbon nanomaterials, including pristine or oxidized single-, double- or multi-walled carbon nanotubes (SWCNTs, DWCNTs, and MWCNTs, respectively), polymer-functionalized CNTs, and GO. In addition, we further converted CNT/PdO and GO/PdO nanohybrids into CNT/Pd and GO/Pd nanohybrids, respectively, by the reduction with NaBH_4_, and tested their use as catalyst in the reduction of 4-nitrophenol. Since the high catalytic activity of PdO has been demonstrated in many previous works [4,5,6,7], the CNT/PdO and GO/PdO nanohybrids could find their important applications in heterogeneous catalysis.

## 2. Results and Discussion

### 2.1. Synthesis

**Figure 1:**
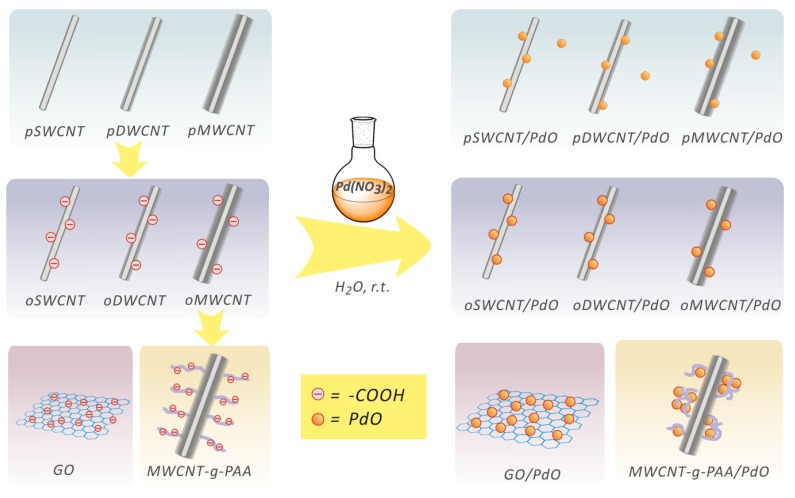
The preparation of CNT/PdO and GO/PdO nanohybrids from Pd(NO_3_)_2_ aqueous solution at room temperature.

Our aqueous solution strategy is schematically outlined in Figure 1. By mixing Pd(NO_3_)_2_ and various kinds of CNTs (or GO) in an aqueous solution at room temperature in air (equation 1), PdO is automatically produced *in situ* along the convex surfaces of CNTs (or the flat surfaces of GO) by chemical reaction (1), resulting in the CNT/PdO (or GO/PdO) nanohybrids. The CNTs samples used here include pristine, oxidized (containing carboxylic groups), and poly(acrylic acid) (PAA)-functionalized CNTs.

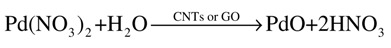



Three different types of CNTs, *i.e.*, SWCNTs, DWCNTs, and MWCNTs, are all examined and show similar results. In addition, control experiments in nitrogen atmosphere are also conducted and the results are the same as those in the presence of oxygen. It is noteworthy that we did not use any additional reducing agent or chemcials, elevated temperature or irradiations in such a process. The spontaneous formation of PdO nanoparticles on the sidewalls of CNTs and GO might be attributed to the redox property [22,23] and electrocatalytic nature [24,25] of CNTs and GO as well as the fact that PdO is the stablest oxide stoichiometry of palladium oxide [26]. This novel method provides a simple and general one-step approach for the growth of PdO nanopartciles with CNTs and GO as the supports. 

### 2.2. MWCNT/PdO nanohybrids

Pristine MWCNTs (pMWCNTs) were refluxed in the mixture of concentrated HNO_3_ and H_2_SO_4_, affording oxidized MWCNTs (oMWCNTs). pMWCNTs have poor dispersion in water, so a long ultrasonic bath treatment is needed before the reaction to help the dispersal of pMWCNTs. oMWCNTs can form stable aqueous solutions upon ultrasonication, since there are many carboxylic acid groups on their surfaces. The Pd(NO_3_)_2_ aqueous solution was added dropwise into the pMWCNTs and oMWCNTs solutions to produce pMWCNT/PdO and oMWCNT/PdO nanohybrids, respectively. Shortly after the adding of the first several drops of the dark brown Pd(NO_3_)_2_ solution into the homogeneous solution of oMWCNTs, numerous flocculent conglomerations appeared, and the brown solution was completely bleached. It is an indication that Pd^2+^ ions in the solution were consumed and PdO nanoparticles were generated on the nanotubes, which made the nanotubes no longer soluble and thus they precipitated from water. When more and more Pd(NO_3_)_2_ solution was added, the color of the reaction solution finally became brown again and did not change, indicating the presence of excess Pd^2+^ ions. After all of the Pd(NO_3_)_2_ solution was added, three samples were taken from the reaction solution at given time (*i.e.*, 1, 10, and 20 h). The samples were washed repeatedly with deionized water and separated by centrifugating.

**Figure 2:**
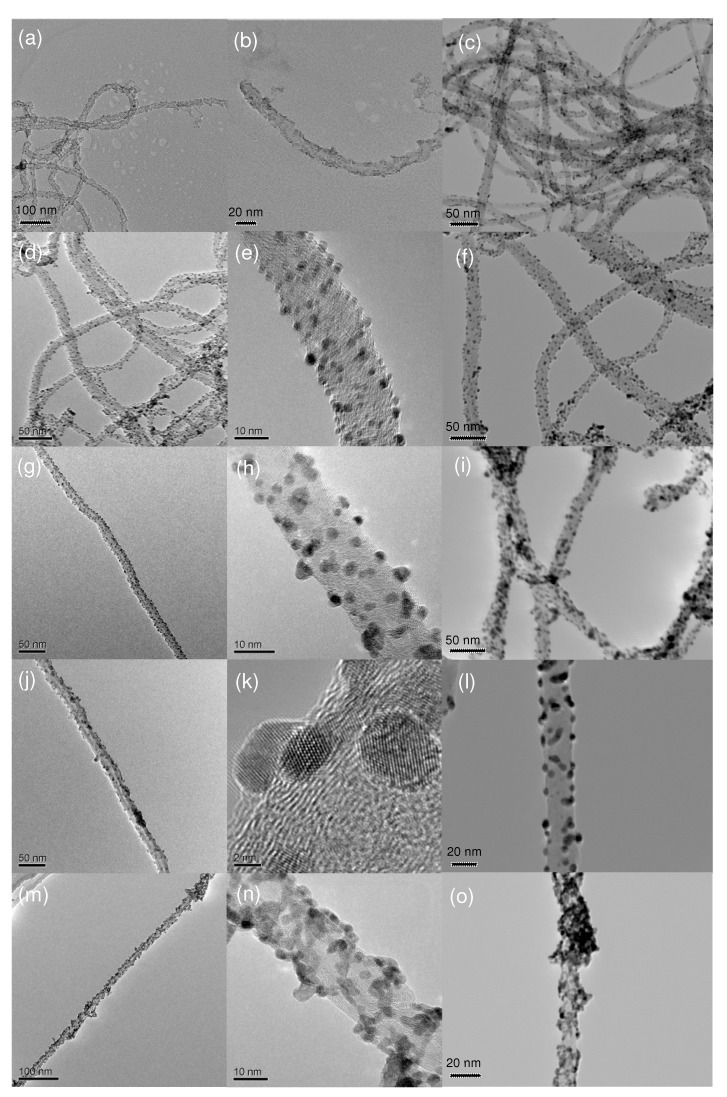
TEM and STEM images of pMWCNT/PdO (a-c), oMWCNT/PdO (d-l) of different reaction time (d-f: 1h, g-i: 10 h, and j-l: 20 h), and MWCNT-*g*-PAA/PdO (m-o). The left and middle columns represent the TEM images, and the right column the STEM images.

The structure and morphology of the resultant samples were observed by transmission electron microscopy (TEM) and scanning transmission electron microscopy (STEM). The typical TEM images of MWCNT/PdO nanohybrids show that both the pMWCNTs and oMWCNTs are evenly coated with PdO nanodots with a uniform size of *ca.* 2–3 nm in diameter (Figure 2a,b,d,e,g,h,j,k). The clear crystalline lattice fringes of the PdO NPs can also be observed in the high-resolution transmission electron microscopy (HR-TEM) image (Figure 2k). The samples with different reaction times exhibit similar sizes of PdO NPs (statistical mean diameters are 2.5, 2.6, and 2.4 nm for the samples of 1, 10, and 20 h, respectively), suggesting that the nucleation and formation of PdO NPs was essentially completed within 1 h, and prolonging the reaction time has little influence on the morphology of MWCNT/PdO nanohybrids. It can be also seen from the TEM images that pMWCNTs and oMWCNTs have different capacities of anchoring PdO NPs: the PdO NPs are nearly all located on the convex suface of oMWCNTs, but a few ones have detached from pMWCNTs and scattered onto the carbon film of the TEM copper grid. This is because pMWCNTs have no carboxylic groups on their inert surfaces, so the adhesion of PdO NPs to the surfaces of pMWCNTs is much weaker than that of oMWCNTs, and the physically adsorbed PdO NPs may drop off the pMWCNTs upon ultrasonication. Besides, the high contrast images of the MWCNT/PdO nanohybrids were obtained by STEM owing to the distinct electron transmission ability of carbon and metal oxide. As can be seen in Figure 2c, f, i, l, plenty of dark spherical nanoparticles were evenly decorated along the grey nanotubes.

**Figure 3:**
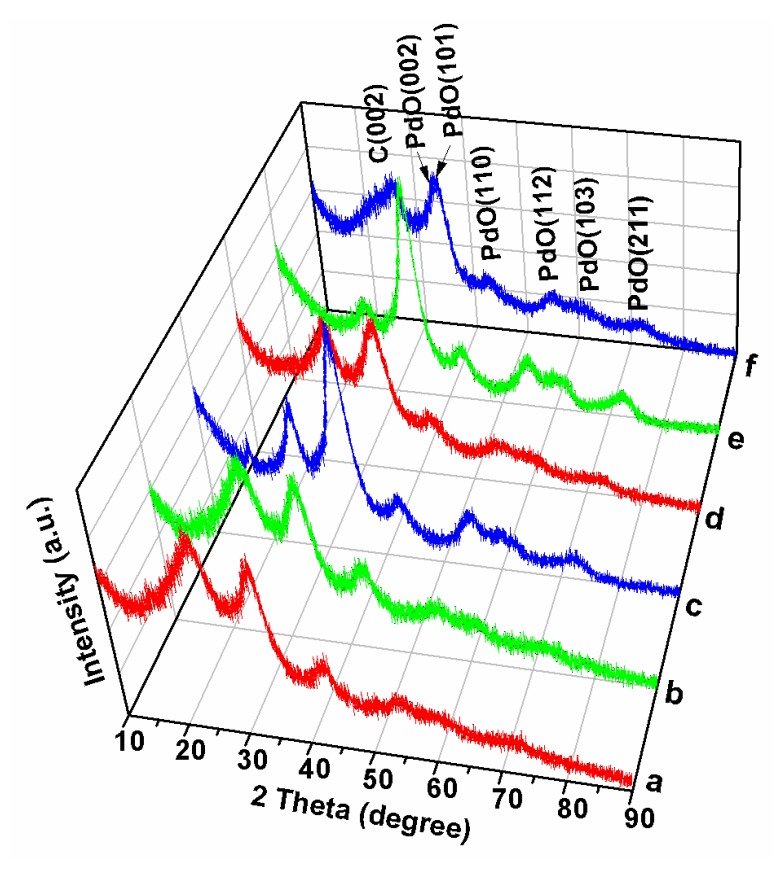
XRD patterns of pSWCNT/PdO (a), pDWCNT/PdO (b), pMWCNT/PdO (c), oSWCNT/PdO (d), oDWCNT/PdO (e), and oMWCNT/PdO (f).

Powder X-ray diffraction (XRD) was used to investigate the phase structure of the obtained MWCNT/PdO nanohybrids. The XRD patterns of pMWCNT/PdO and oMWCNT/PdO nanohybrids are shown in Figure 3c,f. All peaks can be indexed to two phases: hexagonal C (JCPDS Card No.75-1621) with its (002) peak at 25.5°, and tetragonal PdO (JCPDS Card No. 75-584) with its peaks at 2θ 33.6° (facet 002), 34.0° (101), 42.0° (110), 54.9° (112), 60.3° (103), and 71.7° (211). Through the use of the Debye-Scherrer formula on the PdO peaks, the average nanoparticle size for the PdO is estimated to be 2.3 nm for pMWCNT/PdO and 2.2 nm for oMWCNT/PdO, which agrees well with the TEM observations.

**Figure 4:**
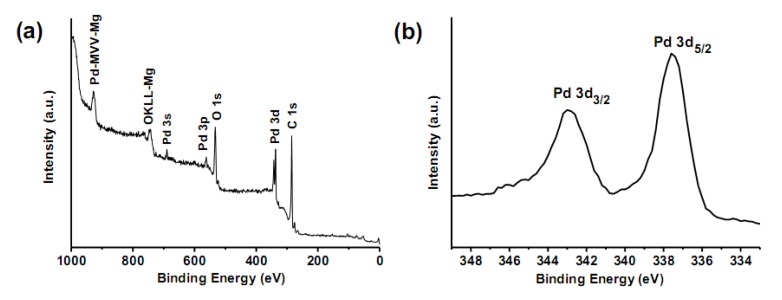
XPS spectra of oMWCNT/PdO: (a) survey of the spectral region from 0 to 1000 eV, and (b) the palladium 3d region.

X-ray photoelectron spectroscopy (XPS) analysis was performed to obtain the information on the electronic state of the surface region of the resulting samples. As shown in Figure 4a, obvious peaks of C 1s, O 1s, Pd 3s, Pd 3p, and Pd 3d are observed in the XPS spectrum of oMWCNT/PdO nanohybrids. 

The enlarged XPS spectrum (shown in Figure 4b) indicates the binding energy (BE) of Pd 3d_5/2_ and Pd 3d_3/2_ at 337.5 and 342.9 eV, respectively, which are coincident with the reported values [27,28]. The Pd 3d_5/2_ peak of PdO NPs shifts to ~1.0 eV higher BE than the reported values for bulk PdO (~336.2–336.5 eV), likely resulting from the substrate-nanoparticle interphase polarization effects [29], initial-state effects (the change of electronic structure on passing from the isolated atoms to the bulk sample) [30], and final-state effects (the differential charging and variations of the relaxation energy) [31]. It has been shown in a previous report [32] that both experiment and theory demonstrated that for the small metal clusters on weakly interacting substrates (have no localized p or d orbitals with BE overlapping those of the metal cluster d orbitals) such as carbon, the metal-support interaction is weak and the shifts are to higher BE for the clusters relative to the bulk metal. So we think that the BE shifts observed in small metal clusters on carbon support are primarily due to the initial-state effects.

**Figure 5:**
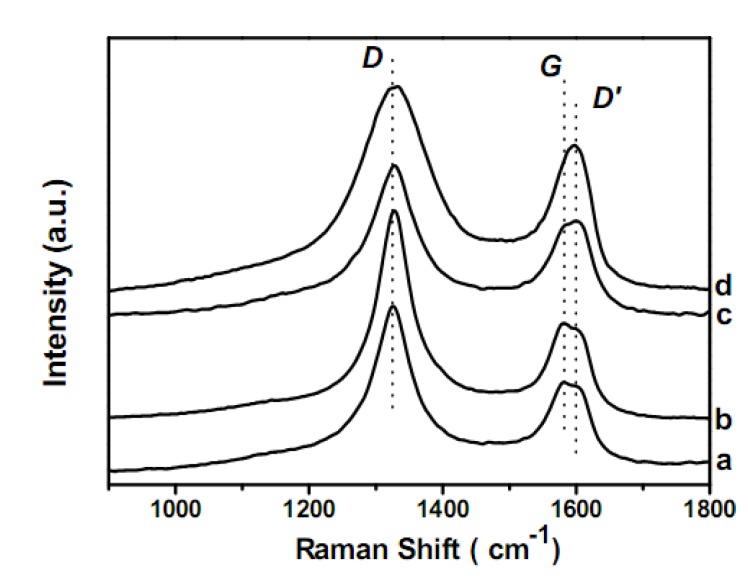
Raman spectra of pMWCNTs (a), oMWCNTs (b), pMWCNT/PdO (c), and oMWCNT/PdO (d).

Raman spectroscopy is a rapid, sensitive and nondestructive analytical technique capable of providing accurate structural and electronic information nanostructures. The Raman spectra of pMWCNTs, oMWCNTs, pMWCNT/PdO, and oMWCNT/PdO recorded at room temperature under ambient pressure are shown in Figure 5. Two distinct absorption bands are clearly observed: the band at ~1,320 cm^-1^ is the disorder mode band (*D* band) that related to the defects and disorder induced modes in the nanotubes, and the band at ~1580 cm^-1^ (*G* band) is attributed to the vibration of sp^2^-bonded carbon atoms in the planar hexagonal graphite lattice, accompanying by *D*’ band at 1,600 cm^-1^ as a shoulder that associated with the disorder induced effect. The *D*- to *G*-band intensity ratio (*I*_D_/*I*_G_) is an approximate indication of the defects and disorder degree in crude CNTs and functionalized CNTs. The band intensity is calculated from the Lorentzian fitting area of the corresponding band in the Raman spectra. The values of *I*_D_/*I*_G_ is 2.06 for pMWCNTs, and it increases to 2.32 for oMWCNTs, revealing that the acid oxidation treatment enhances the defects of pMWCNTs by introducing carboxylic, carbonyl, and hydroxyl groups. The values of *I*_D_/*I*_G_ for pMWCNT/PdO (2.18) and oMWCNT/PdO (3.16) are greater than that of pMWCNTs and oMWCNTs, respectively, which implies the defects and disorder of nanotubes increase after functionalization with PdO NPs.

**Figure 6:**
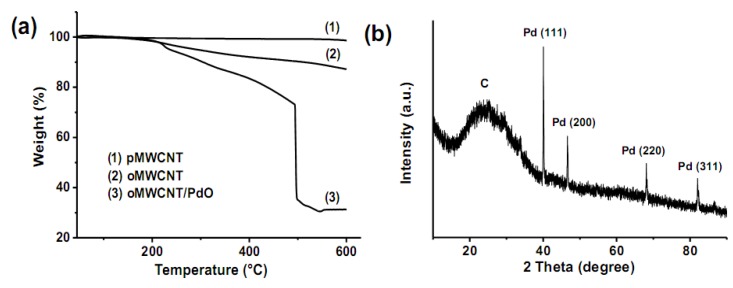
(a) TGA curves of (1) pMWCNTs, (2) oMWCNTs, and (3) oMWCNT/PdO. (b) XRD pattern of the residual powders of oMWCNT/PdO collected after the TGA measurement.

The thermal properties of selected samples are investigated by thermal gravimetric analysis (TGA), and the results are shown in Figure 6a. The weight loss of pMWCNTs below 600 °C is only 1.4 %, but it increases to 12.8% for oMWCNTs due to the decomposition of organic groups on the surfaces of nanotubes. The TGA curve of oMWCNT/PdO nanohybrids slowly decreases at 200–494 °C and sharply decreases at 494–500 °C. This accords with the known fact that PdO is stable at low temperature and decomposes into metallic Pd and oxygen at elevated temperature [33]. The transformation from PdO to Pd is corroborated by the XRD analyses of the same sample after TGA measurement (Figure 6b). It is also worth noting that the PdO NPs decompose at a much lower temperature (~494 °C) than bulk PdO (~790 °C) [34]. This is because carbon materials have reducing properties that will facilitate the reduction reaction, and the nanometer-sized materials usually have a lower melting point than the bulk forms due to their large surface to volume ratio and high surface energy [35]. With a concomitant release of O_2_ upon the decomposition of PdO, the CNTs are probably partially combusted and thus show a weight loss as high as 68.7% at 600 °C.

### 2.3. MWCNT-g-PAA/PdO nanohybrids

**Figure 7:**
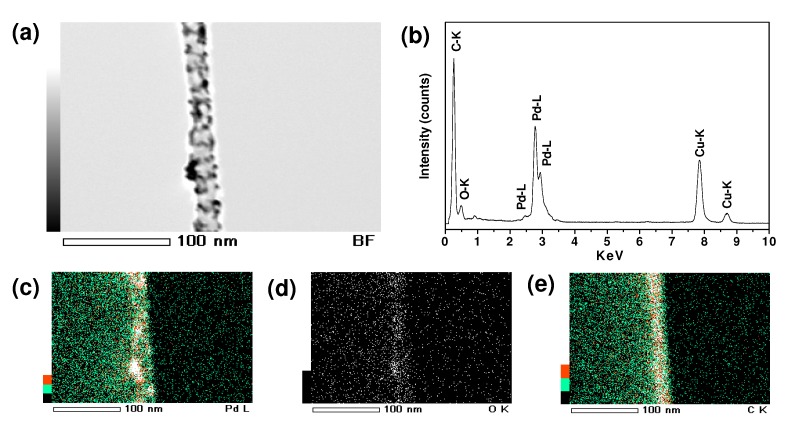
EDS and element mapping of MWCNT-*g*-PAA/PdO: (a) bright field, (b) EDS spectrum, (c-e) palladium, oxygen, and carbon maps.

To control of the morphology of the PdO nanocrystals, poly(acrylic acid)-grafted MWCNTs (MWCNT-*g*-PAA) are also employed here using the same experimental protocol as in the case of MWCNTs. The preparation of MWCNT-*g*-PAA (60 wt % of PAA) has been described previously [36], and involves the surface-initiating atom transfer radical polymerization (ATRP) from the initiating sites previously anchored onto the convex surfaces of MWCNTs. The grafted polymers have a higher density of carboxylic groups, rendering the CNTs excellent dispersibility in a solvent, and the networks of CNTs become individually separated nanotubes. With several drops of PdO aqueous solution added, many flocculent conglomerations formed in the formerly homogeneous aqueous solution of MWCNT-*g*-PAA, but the conglomerations were smaller than those in the cases of MWCNTs. The solution was brown at the end of the reaction, indicating excess Pd^2+^ ions were present. The solid product was then separated by centrifugating and rinsed with deionized water repeatedly. XRD spectrum of the resulting MWCNT-*g*-PAA/PdO nanohybrids shows a similar pattern to the case of oMWCNT/PdO (data not shown here), revealing the crystalline phase structure of PdO NPs. TEM and STEM (Figure 2m-o) display the PdO NPs (~3 nm) are located along the nanotubes and embedded in the grafted polymer layer. The element mapping (Figure 7c-e) also shows the distribution of element Pd and O in the carbon nanotubes is essentially uniform, and matches well with that of PdO NPs shown in the bright field (Figure 7a). Furthermore, the energy dispersive X-ray spectroscopy (EDS) analyses (Figure 7b) indicated that the palladium content of the MWCNT-*g*-PAA/PdO nanohybrids is 40.5 wt %. The results differ from the case of CNTs without grafted polymer in that 1) the density of PdO NPs is higher, 2) the NPs tend to aggregate together, and 3) some big protuberances on the surfaces of nanotubes that consist of many PdO NPs are observed. The morphology difference is largely owing to the strong interaction between PAA polyelectrolyte chains and PdO NPs. The comparison results demonstrate that the polymer functionalization of CNTs will greatly influence the morphology of the PdO NPs in the CNT/PdO nanohybrids.

### 2.4. SWCNT/PdO and DWCNT/PdO nanohybrids

**Figure 8:**
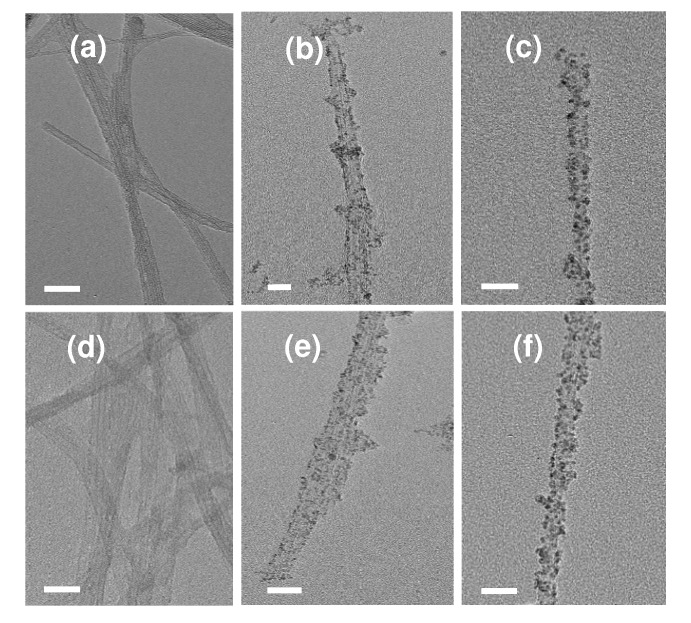
TEM images of oSWCNTs (a), pSWCNT/PdO (b), oSWCNT/PdO (c), oDWCNTs (d), pDWCNT/PdO (e), and oDWCNT/PdO (f). Scale bar: 20 nm.

In order to assess the compatibility of our approach to other types of CNTs, we have also conducted parallel experiments on SWCNTs and DWCNTs. The experimental phenomena are almost the same as those of MWCNTs. The resulting pSWCNT/PdO, oSWCNT/PdO, pDWCNT/PdO and oDWCNT/PdO nanohybrids are characterized by XRD and TEM, and the results are shown in Figure 3a, b, d, e and Figure 8, respectively. 

The XRD analyses confirm the presence of PdO phase in all of these samples. Similarly, the TEM images show that PdO NPs with average sizes of 2.0–2.4 nm are evenly deposited on the pristine and oxidized SWCNTs and DWCNTs bundles. In the TEM of pSWCNT/PdO and pDWCNT/PdO nanohybrids, some isolated PdO NPs can be found on the carbon film of the TEM copper grid due to their weak interaction with inert nanotube surfaces, which is consistent with the case of pMWCNTs. Thus, it can be concluded from the above results that SWCNTs and DWCNTs also have the ability of producing PdO NPs, and different wall thicknesses (single-, double-, and multi-wall) of CNTs have little effect on the forming of CNT/PdO nanohybrids.

Besides the CNT/PdO nanohybrids, our previous work has also tried other transitional metal compounds instead of Pd(NO_3_)_2_ to produce CNT/metal nanohybrids by the same aqueous phase-based synthesis strategy [19]. By using AgNO_3_ aqueous solution, CNT/Ag nanohybrids were obtained from functionalized CNTs (including oSWCNTs, oDWCNTs, oMWCNTs, and MWCNT-*g*-PAA), but pristine CNTs (without oxidation or other treatment) cannot produce metallic Ag particles. By using HAuCl_4_ aqueous solution, CNT/Au nanohybrids were obtained from both pristine CNTs and functionalized CNTs. Interestingly, for AgNO_3_ and HAuCl_4_, the crystals produced by SWCNTs and DWCNTs are all much bigger than those produced by MWCNTs, indicating that SWCNTs and DWCNTs have a higher Ag and Au productivity than MWCNTs, repectively. Surprisingly, SWCNTs, DWCNTs, and MWCNTs have nearly identical productivity of PdO NPs. These different phenomena among Ag, Au and Pd on various types of CNTs can be ascribed to 1) the different standard reduction potentials of Ag^+^/Ag, AuCl^4-^/Au, and Pd^2+^/Pd, which are +0.80, +1.00, and +0.95 V *vs*. standard hydrogen electrode (SHE), respectively [22,37]; 2) the different work functions and Fermi levels of pristine CNTs and functionalized CNTs (e.g., the work functions of pSWCNTs, pMWCNTs, and oMWCNTs are 5.05, 4.95, and 5.4 eV, respectively) [38,39]; and 3) the different stabilities of silver, gold, palladium and their oxides (e.g., Ag_2_O and Au_2_O_3_ have lower thermal and acid stability than PdO).

### 2.5. GO/PdO nanohybrids

**Figure 9:**
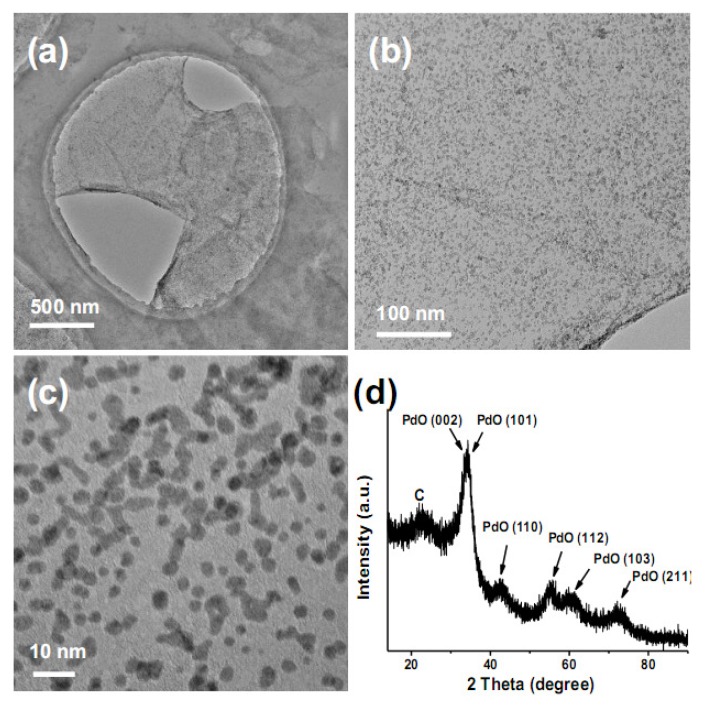
TEM images (a-c) and XRD pattern (d) of GO/PdO.

GO has abundant carboxyl and hydroxyl groups on its surfaces as a result of the oxidation of graphite with strong oxidants. [21] Due to its high hydrophilicity, GO can form homogeneous colloidal suspensions in water upon sonication. By using the same synthesis method and GO instead of CNTs as the starting material, we have also produced graphene oxide/PdO (GO/PdO) nanohybrids. The TEM images in Figure 9a-c show that the surfaces of GO nanosheets are evenly decorated with monodisperse PdO NPs with an average diameter of 2.9 nm. The XRD pattern of the GO/PdO nanohybrids in Figure 9d confirms the presence of PdO crystals. It can be concluded that both the two-dimensional planar surfaces of GO and the one-dimensional tubular surfaces of CNTs are suitable substrates for the producing of PdO NPs, and they have no obvious influence on the morphology or distribution of PdO NPs.

### 2.6. CNT/Pd and GO/Pd nanohybrids

Previous works have shown that PdO can be transformed into metallic Pd by heating above its decomposition temperature [40], or by H_2_ through a solid state reduction process [41]. However, all of these reduction procedures involve elevated temperature, and thus are not suitable for the PdO NPs on the CNTs and GO due to the limited thermal stability of CNT/PdO and GO/PdO nanohybrids. Here we find an alternative way to convert PdO into Pd by the reduction of NaBH_4_ in aqueous phase at room temperature.

**Figure 10:**
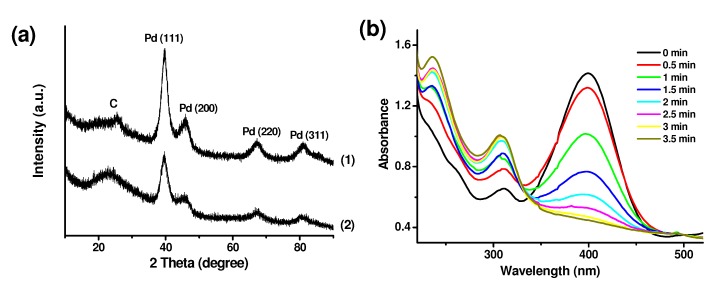
(a) XRD pattern of oMWCNT/Pd (1) and GO/Pd (2). (b) Successive UV-vis spectra for the reduction of 4-nitrophenol into 4-aminophenol with the oMWCNT/Pd catalyst.

The reduction was performed by simply adding the as-prepared CNT/PdO and GO/PdO nanohybrids in NaBH_4_ aqueous solution and stirring for 10 min. The complete transformation of the crystal phase was detected by the changes in the XRD pattern. Figure 10a shows the sample of oMWCNT/PdO and GO/PdO nanohybrids after reduction has a typical XRD pattern of face-centered-cubic (fcc) Pd crystal structure with the diffraction peaks at 39.8°, 46.2°, 67.6°, and 81.4°, corresponding to (111), (200), (220), and (311) planes, respectively. The resulting CNT/Pd nanohybrids exhibit high catalytic activity in the reduction reaction of 4-nitrophenol [42]. Immediately after the adding of CNT/Pd nanohybrids, the UV-vis absorption peak at 400 cm^-1^ that is attributed to 4-nitrophenol quickly reduced and eventually disappeared (Figure 10b). The concomitant appearance of a new peak at 310 nm originates from 4-aminophenol- the reduction product of 4-aminophenol. After separated by centrifigating, the CNT/Pd nanohybrids can be reused in this reaction for at least 10 cycles. Such a strategy provides not only a new method for the generating of Pd from PdO, but also a new way to prepare Pd NPs loaded carbon nanomaterials for use as heterogeneous catalysts.

## 3. Experimental

### 3.1. Materials

Pristine MWCNTs (purity >95%) made by the chemical vapour deposition method were purchased from Tsinghua-Nafine Nano-Powder Commercialization Engineering Centre in Beijing, P.R. China. Pristine SWCNTs (purity of CNTs >90%, purity of SWCNTs >50%, diameter <2 nm, amorphous carbon <5%, special surface area >400 m^2^/g) and DWCNTs (purity of CNTs > 90%, purity of DWCNTs >50%, diameter <5 nm, amorphous carbon <5%, special surface area >400 m^2^/g) were obtained from Shenzheng Nanotech Port (China). GO was prepared from graphite powder (40 μm, Qingdao Henglide Graphite Co., Ltd.) by the modified Hummers method [43], and the details are given in the Supporting Information. Palladium (II) nitrate dihydrate (Pd(NO_3_)_2_•2H_2_O, 99%), 4-nitrophenol (99%), sodium borohydride (NaBH_4_, 96%) were purchased from Sinopharm Chemical Reagent Co., Ltd. and used as received.

### 3.2. Characterizations

Transmission electron microscopy (TEM) and scanning transmission electron microscopy (STEM) studies were performed on a JEOL JEM 2200FS field emission electron microscope (equipped with an energy-dispersive spectrometer, EDS) at 200 kV. X-ray diffraction (XRD) patterns were recorded on a Rigaku X-ray diffractometer D/max-2200/PC equipped with Cu Kα radiation (40 kV, 20 mA). X-ray photoelectron spectroscopy (XPS) measurements were made on a RBD upgraded PHI-5000C ESCA system (Perkin-Elmer) with Mg Kα radiation (*h ν* = 1253.6 eV) at a power of 250 W. Raman spectra were collected on a LabRam-1B Raman spectroscope equipped with a 632.8 nm laser source. Thermal gravimetric analysis (TGA) was carried out on a TA Instruments TGA-2050 thermogravimetric analyzer with a heating rate of 20 °C/min under a nitrogen flow rate of 60 mL/min. UV-visible spectra were recorded using a Varian Cary 300 Bio UV–vis spectrophotometer.

### 3.3. Modification of CNTs

Oxidized CNTs were prepared by refluxing pristine CNTs in the mixture of concentrated H_2_SO_4_ and HNO_3_ (3:1 by volume) for 100 min at 90–133 °C, and then collected by repeated filtration and centrifuging [44]. The poly(acrylic acid)-grafted MWCNTs (MWCNT-*g*-PAA) with PAA contents of 60 wt % were synthesized according to the previous paper [36]. The details are given in the Supporting Information.

### 3.4. PdO support on CNTs and GO

Typically, to a 100 mL flask containing 20 mL of deionized water, CNTs (or GO, 10 mg) were added. After sonication in an ultrasonic bath (40 kHz), the flask was placed on a magnetic stirrer. With stirring at room temperature, a 0.01 M aqueous solution of Pd(NO_3_)_2_ (20 mL) was added dropwise into the flask. After a given time (1 to 20 h), the solid of CNT/PdO (or GO/PdO) was then separated from the mixture by centrifuging and washed repeatedly with deionized water.

### 3.5. CNT/Pd, GO/Pd and catalysis measurements

The as-prepared oMWCNT/PdO (or GO/PdO) (5 m) was added into aqueous NaBH_4_ solution (32 mM, 2 mL) and stirred. After 10 min, the solid of CNT/Pd (or GO/PdO) was collected by centrifuging and washed repeatedly with deionized water. In the standard quartz cuvette with a 1 cm path length, double-distilled water (1 mL), 4-nitrophenol aqueous solution (2 mM, 0.1 mL), and NaBH_4_ in 0.1 M NaOH aqueous solution (32 mM, 1 mL) were added. All solutions were previously deaerated and saturated with N_2_. After the adding of oMWCNT/Pd nanohybrids (1 mg) into the above solution, the absorption spectra were recorded every 30 s in the range of 220–520 nm at room temperature.

## 4. Conclusions

We have successfully synthesized PdO NP decorated CNTs and GO by a simple yet efficient aqueous phase-based “green” method. This method has been demonstrated to be feasible for GO and various kinds of CNTs, including chemically modified or unmodified SWCNTs, DWCNTs and MWCNTs. The EDS, XRD, XPS, Raman, and TGA measuments confirm the generation of PdO nanocrystals on the CNTs and GO. TEM and/or STEM observations show that PdO NPs with diameters of 2–3 nm are produced evenly on CNTs and GO. The parallel experiments show that the reaction time (if longer than 1 h) and different types of CNTs have little influence on the size of the PdO NPs. The density and distribution of the PdO NPs are influenced greatly by different modifications of CNTs (pristine, oxidized or polymer-functionalized), but relatively little by wall thicknesses of CNTs (single-, double-, and multi-walled) or different shapes of carbon nanomaterials (CNTs and GO). The CNT/PdO and GO/PdO nanohybrids are further transformed into CNT/Pd and GO/Pd nanohybrids through the reduction with NaBH_4_, respectively, which show catalytic activity in the reduction of 4-nitrophenol. The novel PdO and Pd NPs decorated carbon nanomaterials have promising applications in heterogeneous catalysis.
